# Evidence-to-decision frameworks: a review and analysis to inform decision-making for environmental health interventions

**DOI:** 10.1186/s12940-021-00794-z

**Published:** 2021-12-08

**Authors:** Susan L. Norris, Max T. Aung, Nicholas Chartres, Tracey J. Woodruff

**Affiliations:** 1 Department of Family Medicine, Oregon Health & Science University, Portland, OR 97239 USA; 2 Program on Reproductive Health and the Environment, Department of Obstetrics, Gynecology & Reproductive Sciences, University of California San Francisco, San Francisco, California USA

**Keywords:** Evidence-to-decision frameworks, Recommendations, Guideline development, Environmental health interventions, Policy, Risk management

## Abstract

**Background:**

Evidence-to-decision (EtD) frameworks provide a structured and transparent approach for groups of experts to use when formulating recommendations or making decisions. While extensively used for clinical and public health recommendations, EtD frameworks are not in widespread use in environmental health. This review sought to identify, compare and contrast key EtD frameworks for decisions on interventions used in clinical medicine, public health or environmental health. This information can be used to develop an EtD framework suitable for formulating recommendations for interventions in environmental health.

**Methods:**

We identified a convenience sample of EtD frameworks used by a range of organizations. We searched Medline for systematic reviews of frameworks. We summarized the decision criteria in the selected frameworks and reviews in a qualitative manner.

**Findings:**

Fourteen organizations provided 18 EtD frameworks; most frameworks focused on clinical medicine or public health interventions; four focused on environmental health and three on economic considerations. Harms of interventions were examined in all frameworks and benefits in all but one. Other criteria included certainty of the body of evidence (15 frameworks), resource considerations (15), feasibility (13), equity (12), values (11), acceptability (11), and human rights (2). There was variation in how specific criteria were defined. The five identified systematic reviews reported a similar spectrum of EtD criteria.

**Interpretation:**

The EtD frameworks examined encompassed similar criteria, with tailoring to specific audience needs. Existing frameworks are a useful starting point for development of one tailored to decision-making in environmental health.

**Funder:**

JPB Foundation.

**Supplementary Information:**

The online version contains supplementary material available at 10.1186/s12940-021-00794-z.

## Introduction

It is widely recognized that environmental pollution is an important determinant of health and that interventions, in particular policy recommendations, are a key means by which population health can be improved. The formulation of trustworthy and impactful recommendations and policies on environmental health interventions is a complex task, which requires the identification of all relevant data and evidence, their critical appraisal and synthesis and translation into a recommendation or policy. The processes and methods for hazard identification and risk assessment of environmental substances based on systematic reviews of the evidence have advanced considerably in the last decade [1–4]. These approaches have been largely based on methods developed for clinical medicine [5], which have been expanded to encompass public health interventions [6], diagnostic test accuracy and impact [7], coverage decisions [8, 9], and health technology assessments (HTA) [10], among others.

The final step in the process of formulating evidence-informed recommendations and policies involves the translation of data and evidence on various decision factors into an explicit recommendation or policy. Evidence-to-decision (EtD) frameworks provide a structured and transparent approach for groups of technical experts or policy-makers to accomplish this step [6, 11]. These frameworks include explicit criteria which the group considers individually and in aggregate focused on the relative benefits and harms, as well as other considerations. Such frameworks can facilitate: i) consideration of all relevant criteria in the decision-making process; ii) examination of the pros and cons of each intervention option; iii) presentation of relevant evidence for each criterion; iv) identification of the reasons for any disagreement within the expert group; v) transparent reporting of the decision-making process; and vi) crafting of the rationale statement for each recommendation. Populated EtD frameworks can also facilitate implementation by assisting the end-user in understanding how and why specific recommendations were made, and by providing data and evidence on each decision criterion which may facilitate local adoption or adaptation [12].

In environmental health, once hazards are identified and risks assessed, organizations may want to examine mitigating and prevention interventions and make recommendations and policies based on systematic reviews of research evidence and other data and information. An EtD framework suitable for environmental health interventions will facilitate this process. However, such frameworks have not been widely used in this field.

The objectives of this review were to identify, compare and contrast key EtD frameworks for interventions in clinical medicine, public health and environmental health and to summarize the main decision criteria across these frameworks. The identification of these criteria will inform the development of an EtD framework for use in formulating recommendations regarding mitigating and prevention interventions related to exposure to harmful substances in the environment.

## Methods

In order to identify relevant, existing EtD frameworks, we took two approaches: a search of the peer-reviewed literature for systematic reviews of EtD frameworks, and identification of frameworks used by a range of organizations which make decisions or formulate recommendations in clinical medicine, or public or environmental health.

### Scope

The focus of this paper is on the substantive criteria for decision-making with respect to interventions, including both normative criteria (what should be done) and feasibility criteria (what can be done) [13]. Criteria related to the process of decision-making are beyond the scope of this work.

There are an increasing number of organizations which use systematic and transparent approaches to synthesize evidence in environmental health for hazard identification [14], risk assessment [15], and for the synthesis of the benefits and harms of interventions [16]. These organizations were not included in the current analysis because they do not make recommendations on interventions to mitigate the effects of harmful exposures.

### Bibliographic database search and article screening

To identify relevant EtD frameworks, we searched Medline via PubMed for systematic reviews of EtD frameworks, templates or tools published in English and indexed in the 10-year period up to 7 January 2021. The complete search strategy is found in Annex 1. We also solicited the advice of guideline development experts for any additional potential citations. Publications were included if they were systematic reviews of frameworks, tools or templates for formulating decisions or recommendations, or for priority setting related to the use of interventions (including diagnostic tests) in clinical medicine, public health, or environmental health. The included frameworks focused on population-level interventions; decision tools for the provider-patient interaction and patient decision aids were excluded. Frameworks which focused exclusively on economic considerations were also excluded. The setting for both decision-making and implementation of recommendations was not restricted.

Two persons (SLN and MTA) screened the titles and abstracts, and full-text versions were retrieved for studies which potentially fulfilled inclusion criteria. Consensus was achieved between the two reviewers for final inclusion in the review.

### Search for EtD frameworks used by key organizations

In addition to the systematic review described above, we examined a broad range of organizations including clinical and public health guideline development groups in academia and the private sector, healthcare provider professional organizations, governmental agencies and international organizations. Given the purpose of this review, we focused particularly on organizations that work in the field of environmental health. Organizations were identified based on author knowledge of prominent guidelines in public health and clinical medicine, by snowballing (reviewing the origin of or basis for identified frameworks), and by conferring with a broad international network of guideline developers based on our experiences and contacts.

We used this pragmatic approach for several reasons. First, because of the dominance of the GRADE (Grading of Recommendations Assessment, Development and Evaluation) EtD framework over the last 15 years [5, 11, 17], many organizations either use GRADE or a modification thereof. Thus, a more exhaustive search was unlikely to yield novel or unique frameworks. Second, because the methods used by guideline development organizations are infrequently published in the peer-reviewed literature, an extensive hand search of the grey literature including organizational web-sites would have been required, which was infeasible.

### Data extraction and synthesis

For each systematic review identified in our search, a single author (SLN) extracted key information describing the focus of the review, time period searched, and the main findings, including an overview of the criteria identified across the included frameworks. We did not extract data on each individual framework included in each review.

For the EtD frameworks used by key organizations, we extracted data on organizational characteristics, the methods used to develop the framework, the funders and any declared interests of the developers of the framework, how evidence should be used to inform each criterion in the framework, quality assessment of individual studies and of the body of evidence, the specific EtD criteria, and the types of conclusions or recommendations formulated. One author extracted these data (SLN) and a second checked them (MTA); disagreements were discussed and consensus reached. Data were extracted into a template in Excel (Microsoft Corporation, Redmond WA, USA).

For the key organizations, the primary decision criteria for each EtD framework were extracted from the main published reports, and specifically from the identified template or list of criteria. Criteria which are mentioned only in the narrative text accompanying a framework were not extracted unless the text suggested that they were consistently applied in decision making.

Data were summarized in a narrative, qualitative manner. Given that the included frameworks were a convenience sample, descriptive statistics and statistical comparisons are not meaningful. Quality assessment of the frameworks was not performed as there is no standard for such an assessment.

### Role of the funding source

The study funder (JPB Foundation) had no role in the study design; data collection, analysis or interpretation; in writing the report; or in the decision to publish.

## Results

### Identified systematic reviews of EtD frameworks

The bibliographic database search for systematic reviews of EtD frameworks yielded 399 citations, of which four fulfilled inclusion criteria [7, 10, 18, 19] (Table 1, Annex 2 and 3). One additional study which fulfilled inclusion criteria was identified by the co-authors [8]. Each of these reviews included a cohort of EtD frameworks which the authors had systematically identified, with a particular focus: multicriteria decision analysis in HTA [10], vaccine adoption into national programs [18], diagnostic tests [7], decision making in local low-income settings [19], and frameworks for informing health system coverage decisions [8]. Each review summarized the main EtD criteria identified across their included, individual frameworks, and these main criteria are presented in Table 1. All five reviews included the balance of benefits and harms and consideration of resource use or cost-effectiveness. Only the review by Mustafa and colleagues [7] included an assessment of certainty of evidence. Considerations of equity, acceptability and feasibility were included in only two reviews [10, 18]. The EtD criteria outlined in these five reviews generally corresponded to the main criteria in the EtD frameworks of the selected key organizations (see Results section 2).

**Table 1:** Systematic reviews of evidence-to-decision frameworks: review characteristics and key findings. Decision criteria are the main criteria that the review authors identified across the frameworks which were included in their review

	Citation	Focus	Years searched; databases	Findings	Decision criteria	Comments
**1**	Baltussen et al. 2019 [10]	Systematic review of MCDA and related terms; focused on HTA: included only studies that included economic analyses	1990 to September 2018; Medline only	*n* = 36 studies; categorized these as qualitative (*n* = 1 study), quantitative (*n* = 35), and MCDA with decision rules (*n* = 0); provided list of included studies but no other details	Criteria for MCDA: 1. Effectiveness 2. Severity of disease 3. Disease of the poor 4. Cost-effectiveness	Search strategy focused only on MCDA and studies that included economic analyses; no data provided on the 36 individual studies
**2**	Burchett et al. 2012 [18]	Systematic review of the literature on national decision-making about adoption of new vaccines into national immunization programs	Through March 2010; Medline and multiple other databases; multiple languages	*n* = 21 unique frameworks	Nine broad categories of criteria: 1. Importance of the health problem (eg disease burden) 2. Effectiveness and safety of the vaccine 3. Programmatic considerations 4. Acceptability 5. Accessibility, equity and ethics 6. Financial/economic issues 7. Impact of vaccination 8. Consideration of alternative interventions 9. Decision-making process	Provides list of domains and sub-domains (Table 2); for vaccine context
**3**	Morgan et al. 2018 [8]	Systematic review of EtD frameworks focusing on decision-making about whether or not to pay for a new healthcare intervention (e.g., test, treatment, or procedure)	2013–2015; multiple databases, English only	*n* = 25 frameworks, each with a set of decision criteria	Variable across the 25 frameworks. Developed a new framework, built on GRADE EtD, including: 1. Burden of disease 2. Benefits and harms 3. Values and preferences 4. Resource use 5. Equity 6. Acceptability 7. Feasibility Modifications included adding limitations of alternative technologies considerations in use (expanding benefits and harms) and broadening acceptability and feasibility constructs to include political and health system factors.	Started witih GRADE for clinical interventions, modified it for coverage/payer decision-making; did not examine the EtD criteria for all 25 identified frameworks.
**4**	Mustafa et al. 2017 [7]	Systematic review to identify tools for assesssing the quality of evidence and the strength of recommendations related to diagnostic strategies and tests in health care	1996 to June 2012; Medine, Embase	Identifed 29 tools and 14 modifications	Over all tools examined, domains to assess strength of recommendations: 1. Quality of evidence 2. Patients and populations beliefs 3. Cost and resources 4. Balance of benefits and harms/burden	Focus on diagnostic tests only; Table 3 includes categories for EtD criteria (with various sub-domains or synonyms)
**5**	Wickremasinghe 2016 [19]	Systematic review of processes and tools for local decision-making in LMIC using information and evidence from health systems data	Search dates NR; published 2016; 14 databases searched	*n* = 10 studies describing the approach of tools for decision--making; includes case studies and 1 realist evaluation.	Not explicitly summarized. Frameworks are reported to include priorization, and estimates of budget and impact from local data.	This study focuses on decision-making in a specific context, using local data. Includes rather narrowly focused decision criteria

Abbreviations: *EtD* evidence-to-decision; *GRADE* Grading of Recommendations, Development and Evaluation; HTA, health technology assessment; *MCDA* multi-criteria decision analysis; *n* number of studies; *NR* not reported

### EtD frameworks used by key organizations

Fourteen organizations that use EtD frameworks for recommendation formulation or decision-making were examined in detail (Table 2). One organization, the GRADE Working Group, is an informal network of individuals from a broad range of organizations, including academic institutions, national and international guideline development agencies, and healthcare provider organizations, among others [42]. The GRADE Working Group does not, itself, publish guidelines, but rather develops processes and methods for use by other organizations which develop guidelines. The other 13 organizations develop guidelines for specific audiences and with a clearly defined scope or set of topics: five are agencies of national governments [25, 31, 33, 35, 39], two are related to the World Health Organization (WHO) [37, 38], one is a U.S. State agency (California Environmental Protection Agency (CalEPA) [22]) and the remainder are non-governmental organizations or academic groups [2, 21, 24, 27, 28].

**Table 2:** Evidence-to-decision frameworks: Characteristics of the organizations that developed the frameworks

	Organization*	Type of organization^**a**^	Target audience; Goal	Year established; current version	Methods for development of the EtD framework	Funder^**b**^ Declarations of interest^**c**^	Use and sources of evidence to inform EtD criteria	Assessment of the quality/certainty of the body of evidence	Names for recommendation or evaluation	No recommendation	Research or knowledge gaps	
1	**Advisory Committee on Immunization Practices (ACIP) (US Centers for Diseased Control and Prevention)** [20]	“US Federal advisory committee that provides expert advice to the Director of CDC and the Secretary of the US Department of Health and Human Services in the form of recommendations on the use of vaccines and related agents for control of vaccine-preventable diseases”	Public health programs in the US; health care providers and persons in the US civilian population; To develop recommendations on how to use vaccines to control disease in the US	Established 1964; EtD framework updated June 2018	Result of expert meeting Feb 2018; adapted from GRADE framework	NR Authors report no conflicts of interest	Systematic reviews of the evidence on benefits and harms	GRADE system	“Recommendations will be communicated in the framework in one of three categories: 1) ACIP recommends vaccination for all persons in an age group or a group at increased risk for vaccine-preventable disease; 2) ACIP does not recommend the use of a vaccine; or 3) the ACIP recommendation relies upon guidance of the clinician in the context of individual clinician-patient interactions to determine whether or not vaccination is appropriate for a specific patient.”	“In some instances (e.g., when additional information is needed), ACIP might not make a recommendation, and this option is also reflected in the EtR framework separately.”	“ACIP workgroups should identify research needs and, if appropriate, prioritize them. In formulating research needs, workgroups should be as specific as possible about what is needed and why. One format is EPICOT (ref: Brown P et al. BMJ 2006;333:804–6):”	
2	**Breast Cancer Prevention Partners (BCPP)** [21]	Non-governmental organization in the US	State policy-makers, health systems and healthcare providers; Prevention of breast cancer; focus on the intersection of breast cancer and environmental health	Established 1992; Methods published September 2020	Expert committee, input from community representatives and other stakeholders	California Breast Cancer Research Program NR	Systematic reviews of the evidence on interventions	NR	NR	NR	Research gaps highlighted for each risk factor	
3	**California Environmental Protection Agency (CalEPA)** [22]	US state governmental agency	Policy-makers and regulators in the State of California, USA; To restore, protect and enhance the environment, to ensure public health, environmental quality and economic vitality	Agency established 1991 A Guide to Pesticide Regulation, updated in 2017	NR	NR NR	Systematic reviews for risk assessment	NR	NR	NR	Discusses authorization of agents in the context of research	
	**California Environmental Protection Agency (CalEPA)** [23]	US state governmental agency	Alternative Analysis analysts, preparers, practitioners, and responsible entities; To provide tools, information sources, and best practice approaches to help conduct Alternatives Analysis; challenges “responsible entities to reduce or eliminate toxic chemicals in the products consumers buy and use.... To identify Priority Products containing Chemicals of Concern, and for responsible entities to identify, evaluate, and adopt better alternatives.”	Agency established 1991 Alternative Analysis Guide, version 1.1, July 2020	NR	NR NR	“Relevant factors” for alternatives analysis “can be quantified by available information or based on qualitative information”	Uncertainty analysis performed for individual factors assessed (sensitivity analysis or scenario analysis); no recommendation for quality of the body of evidence	NR	NR	NR	
4	**Evidence and Values Impact on DEcision Making (EVIDEM)** [24]	Developed by a group of authors at private and academic institutions	Variable depending on decision maker; To provide a practical framework to facilitate decision making in a variety of contexts and to enhance the communication of decisions	Established 2006; first published 2008; Current framework: 2018	Review of the literature and or decision-making processes in use; identification of the steps and components of decision-making processes; framework developed by the study authors with input from thought leaders and stakeholders	“The publication costs for this article were funded by Mark O’Freil, the Brinson Foundation, and the Payne Family Foundation”; “No sources of funding were used to conduct this study and internal sources of support for the study were provided by the WSB and BioMedCom Consultants.” Authors declare no completing interests	Relevant evidence is collected and assessed	“Quality of evidence” is assessed using bespoke tools based on existing tools, tailored to each type of evidence; sub-criteria include relevance, validity, completeness of reporting, type of evidence, and consistency.	Varies across end-users of this framework	NR	NR	
5	**Grading of Recommendations, Assessment, Development and Evaluation (GRADE) (clinical - individual or population perspective )**[11, 17]	Consortium of academics and other guideline stakeholders	Varies with the organization/entity using the GRADE system; To standardize assessment of the certainty (quality) of a body of evidence and the formulation of recommendations	Established 2000; Methods continuously updated; EtD framework published 2016	Started with GRADE Working Group approach (Guyatt 2008); iterative process; included brainstorming, feedback from stakeholders, application to recommendations and decisions, user testing (Alonso-Coello BMJ 2016.Introduction)	European Commission Authors report no conflicts of interest	Systematic reviews, primary research, expert opinion; systematic review preferred depending on the criteria	GRADE system	Strong, weak/conditional/discretionary, for or against the intervention	Possible when “pros and cons of the intervention or option and the comparison are so closely balanced that the panel is not prepared to make a weak recommendation in one direction or the other.” and when “there is so much uncertainty that the panel concludes… that a recommendation would be speculative.”	Rcommended when: i) there is insufficient evidence supporting an intervention for a guideline panel to recommend the intervention’s use; ii) further research has a large potential for reducing uncertainty about the effects of the intervention; and iii) further research is deemed good value for the anticipated costs.	
	**GRADE (coverage decisions)** [9]	Consortium of academics and other guideline stakeholders	Third-party payers (public or private) for the purpose of deciding whether and how much to pay for drugs, tests, devices or services and under what conditions; To standardize assessment of the certainty (quality) of a body of evidence and the formulation of recommendations	Established 2000; Methods continuously updated; EtD framework published 2017	Iterative process: brainstorming workshops, consultation with advisory group, user testing, feedback, application to different types of coverage decisions	European Commission One author reports having received funding ffrom the pharmaceutical industry; all other authors report no conflicts of interest	Systematic reviews, primary research, expert opinion; systematic review preferred depending on the criteria	GRADE system	Not covering, coverage only in the context of research, covering with price negotiation, restricted coverage, and full coverage	GRADE clinical EtD guidance likely applies	GRADE clinical EtD guidance likely applies	
	**GRADE (health system and public health decisions)** [6]	Consortium of academics and other guideline stakeholders	Population or health system; specific population perspective depends on the nature of the decision; e.g., could be societal or governmental; To standardize assessment of the certainty (quality) of a body of evidence and the formulation of recommendations	Established 2000; Methods continuously updated; EtD framework published 2018	Iterative process based on the GRADE clinical EtD: brainstorming workshops, consultation with stakeholders, survey of policy-makers, experience with policy briefs, applied the framework to examples, conducted workshops, observed guideline panels using the framework, conducted user testing	European Commission Authors report no conflicts of interest	Research evidence (“information derived from studies that used systematic and explicit methods”); “additional considerations include other evidence such as routinely collected data, and assumptions and logic”	GRADE	Strong, weak/conditional/discretionary, for or against the intervention	GRADE clinical EtD guidance likely applies	GRADE clinical EtD guidance likely applies	
6	**Guide Community Preventive Services (US Centers for Disease Control and Prevention)** [25, 26]	Independent body of experts, funded by the US government and supported by the US Centers for Disease Control and Prevention	Policy-makers at the state or community level, or in community or healthcare organizations, businesses, or schools; To improve health or prevent disease	Established 1998; Methods updated 2017 (unpublished)	Review of the US Preventive Services Task Force methods, input from experts in systematic reviews, the Task Force, and other external advisors	NR NR	Systematic reviews of benefits and harms	Strong, sufficient, insufficient strength of evidence based on quality of execution (study limitations), suitability of study design, number of studies, consistency, meaningfulness of effect size	Recommend, recommend against, insufficient evidence	Yes, “insufficient evidence”, i.e. unable to determine effectiveness	Each chapter includes research and knowledge gaps focusing on effectiveness, applicability in other populations, economic consequences, implementation barriers, and opportunities to improve technical efficiency	
7	**Institute for Clinicaland Economic Review (ICER)** [27]	Independent, non-profit research organization based in the US	Health system managers, policy makers, payers “evaluates medical evidence and convenes public deliberative bodies to help stakeholders interpret and apply evidence to improve patient outcomes and control costs.”	Established 2006: Methods updated Oct. 2020	The initial framework was developed with input from a multi-stakeholder workgroup; followed by national public comment, review and feedback by a broad range of stakeholders.	ICER (which receives its funding from government grants and non-profits foundations); a separate policy program is funded in part by health insurers and other industries NR	Systematic reviews of comparative effectiveness	Systematic reviews of comparative effectiveness include an assessment of certainty of the body of evidence.	Provide assessment as to an intervention’s “value for money”.	NR	Consider future research needs	
8	**International Society for Pharmacoeconomics and Outcomes Research (ISPOR)** [28–30]	Non-profit, multidisciplinary, multistakeholder professional organizationin pharmacoeconomics and outcomes research	Variable depending on who is making decisions To assess the value of new technologies (value is defined from an economic perspective and includes “gross value” (what individuals or others acting on their behalf would be willing to pay to acquire more health care or other goods or services), and “opportunity cost” (what benefits or other resources they are willing to forgo to obtain them))	Established 1995; 2018	Task force appointed by ISPOR, with input from advisory board and stakeholder panel; reviewed existing examples of value assessment frameworks; through disussion and with feedback and review, arrived at final framework .	NR NR	Data are used to measure the performance of alternatives; sources include systematic reviews, modelling, expert opinion, and other approaches as appropriate.	MDCA includes an “uncertainty analysis to understand the level of robustness of the MCDA results”	MDCA results in an assessment of the “total value” of the alternatives under consideration.	NR	NR	
9	**National Institute for Health and Care Excellence (NICE); focus on guidelines** [31, 32]	UK government organization; a non-departmental public body that provides national guidance and advice to improve health and social care in England.	Individual healthcare providers; local authorities, commissioners and managers; other providers of health and social services; To produce evidence-based recommendations on a range of topics, including prevention and management of specific considtions, improving health, managing medicines, providing social care and support, and planning services for communities	Established 1999 Methods guidance published 2014, updated October 2018	“The processes and methods described in this manual are based on internationally recognized standards, and the experience and expertise of the teams at NICE, the contractors..., NICE committee members and stakeholders. They are based on internationally accepted criteria of quality...and primary methodological research and evaluation undertaken by the NICE teams. They draw on the Guideline Implementability Appraisal tool to ensure that recommendations are clear and unambiguous, making them easier to implement.”	NR NR	Systematic reviews of the evidence; “colloquial evidence” can be included (e.g. expert testimony); for economic analyses: systematic review of existing models; may perform de novo model	Individual study quality assessed according to study design; the certainty or confidence in the findings should be presented at outcome level using GRADE or GRADE-CERQual; body of evidence for each outcome is high, moderate, low, very low “certainty or confidence of evidence”; it integrates a review of the quality of cost-effectiveness studies... it does not use ‘overall summary’ labels for the quality of the evidence across all outcomes: “strength of evidence (reflecting the appropriateness of the study design to answer the question and the quality, quantity and consistency of evidence)”: classified as no, weak, moderate, strong or inconsistent evidence	NICE uses the wording of recommendations to reflect the strength of the evidence (e.g. “offer, advise, refer versus consider”)	“If evidence of efficacy or effectiveness for an intervention is either lacking or too low quality for firm conclusions to be reached, the committee.... may: make a ‘consider’ recommendation based on the limited evidence... decide not to make a recommendation and make a recommendation for research... recommend that the intervention is used only in the context of research... recommend not to offer the intervention.”	Include recommendations for research; “The committee should select up to 5 key recommendations for research that are likely to inform future decision-making (based on a systematic assessment of gaps in the current evidence base).”	
10	**Navigation Guide** [2, 16]	Non-profit collaboration between governmental and non-governmental (including academic) organizations in the US and Europe	Clinicians,policy-makers, professional societies, health care organizations, goernment agencies making prevention-oriented guidelines To provide a methodology for evaluating the evidence and to support evidence-basesd decision-making in environmental health	Published 2011 Current version 2014	Collaborative process among clinicians, systematic review and guidelines experts, statistics, epidemiology, and environmental health scientists; based on GRADE	For 2009–2013, support for the development and dissemination of the Navigation Guide methodology was provided by the Clarence Heller Foundation, the Passport Foundation, the Forsythia Foundation, the Johnson Family Foundation, the Heinz Endowments, the Fred Gellert Foundation, the Rose Foundation, Kaiser Permanente, the New York Community Trust, the Philip R. Lee Institute for Health Policy Studies, the Planned Parenthood Federation of America, the National Institute of Environmental Health Sciences ... and U.S. EPA STAR grants. Authors report no conflicts of interest.	Systematic reviews of the evidence on the risks to human health of exposure to chemicals, and the effects of prevention and mitigating interventions	The quality of individual studies and the overall body of evidence is rated, including for human and animal data	NR (Statements about the health risks of substances include: known to be toxic, probably toxic, possibly toxic, not classificable, or probably not toxic.)	NR	NR	
11	**Scottish Intercollegiate Guideline Network (SIGN)** [33, 34]	Supported by the Scottish government, but with editorial independence	Health and social care professionals, patients; To understand and use medical evidence to make decisions about healthcare, reduce unwarranted variations in practice, make sure patients get the best care available, improve healthcare across Scotland	Established 1993; Handbook first published 2008; Current version: November 2019	Based on 2013 GRADE/DECIDE work	Core funding for SIGN activities comes from Healthcare Improvement Scotland NR	Systematic reviews of the evidence	GRADE system	Strong recommendation against; conditional recommendation against; recommendation for research and possibility conditional recommendation for use restricted to trials; conditional recommendation for; strong recommendation for	NR	Include recommendations for research	
12	**US Preventive Services Task Force** [35, 36]	Independent body of experts, funded by the US government	Primary care clinicians, also policy-makers, payers, patients; Tp provide recommendations for preventive care for general, primary care populations in the US who are asymptomatic with respect to the condition addressed by the intervention	Established 1984; Procedure manual Dec 2015	Developed by the Methods Working Group of the USPSTF using an iterative process based on the methods literature, international standards and practices; approved by the Task Force	NR NR	Systematic reviews of benefits and harms; sometimes on contextual questions also	Assessment of certainty across the analytic framework, where certainty is “the likelihood tha thte USPSTF assessment of the net benefit of a preventive service is correct”; “assessing the certainty of evidence requires a complex synthesis of all evidence across the entire analytic framework” in order to determine if “the results observed in the indivudal studies in the body of evidence would be expected when the intervention is delivered to asymptomatic persons by providers in US primary care settings”.	Grades (or strength) of recommendations: A. The USPSTF recommends the service. There is high certainty that the net benefit is substantial. B The USPSTF recommends the service. There is high certainty that the net benefit is moderate, or there is moderate certainty that the net benefit is moderate to substantial. C. The USPSTF recommends selectively offering or providing this service to individual patients based on professional judgment and patient preferences. There is at least moderate certainty that the net bene☐t is small. D. The USPSTF recommends against the service. There is moderate or high certainty that the service has no net bene☐t or that the harms outweigh the benefits.	I Statement. The USPSTF concludes that the current evidence is insufficient to assess the balance of benefits and harms of the service. Evidence is lacking, of poor quality, or conflicting, and the balance of benefits and harms cannot be determined.	Reports on evidence gaps for each clinical preventive service the Task Force reviews; and there is an annual report to congress that focuses on evidence gaps as well	
13	**World Health Organization (WHO) - guidelines** [37]	United Nations agency	National and local policy makers and program managers; To prevent disease and promote health	Established 1948; Handbook for guideline development, 2nd edition: 2014	Adopted directly from the then-current (2014) GRADE approach	The Bill & Melinda Gates Foundation NR	Systematic reviews of benefits and harms and other considerations as indicated	GRADE system	Strong, conditional, for or against	No explicit guidance provided; not prohibited	Added as a requirement in 2019; specific methods and guidance under development	
14	**WHO-INTEGRATE** [38]	Developed for WHO; applies to any entity making public health or health system guidelines	National and local policy makers and program managers; To prevent disease and promote health	Publshed 2019	i) an analysis of WHO’s norms and values; ii) a systematic review of EtD criteria in clinical care and public health; iii) key informant interviews; iv) application to completed WHO guidelines; v) focus groups; vi) peer review; and vii) the development of guidance and prompts for completing the EtD.	Funding provided by the World Health Organization Department of Maternal, Newborn, Child and Adolescent Health through grants received from the United States Agency for International Development and the Norwegian Agency for Development Cooperation One author is a WHO employee; two authors are members of the GRADE Working Group	Evidence gathered as needed to inform key EtD considerations; systematic reviews for key criteria	No specific guidance provided but an assessment is recommended	No specific guidance provided	NR	NR	

Abbreviations: *EtD* evidence-to-decision; *GRADE* Grading of Recommendations, Development and Evaluation; *MCDA* multi-criteria decision analysis; *NR* not reported; *WHO* World Health Organization

Footnotes:

(^a^) Organization that developed the framework.

(^b^) Funder(s) for the development of the framework.

(^c^) Declaration of interests of the developers of the framework.

Two organizations focus primarily on clinical care: the Scottish Intercollegiate Guideline Network (SIGN) [34] and the U.S. Preventive Service Task Force (USPSTF) [36]. The Guide to Community Preventive Services (GCPS) focuses on interventions aimed at groups, communities or health systems [26] and WHO and WHO-INTEGRATE primarily on public health interventions [37, 38]. Three organizations are oriented to health technologies, with a prominent focus on economic evaluations and resource considerations [24, 27, 28]. The UK National Institutes for Health and Care Excellence (NICE) examines a broad range of clinical, public health, and social interventions [32]. The Advisory Committee on Immunization Practices (ACIP) focuses exclusively on vaccine recommendations for U.S. populations [20]. Breast Cancer Prevention Partners (BCPP) [21], CalEPA [22, 23], and the Navigation Guide [2, 16, 40] focus on the human health effects of hazardous substances in the environment.

#### Development process and expertise

For the organizations that developed de novo EtD frameworks and described the process for developing them, all used an iterative approach based on an examination of other organizations, with input from experts in guideline methods and evidence synthesis (Table 2). The most comprehensive approach was taken by Rehfuess and colleagues [38] in development of the WHO-INTEGRATE framework. Their approach included development of a theoretical framework, a review of WHO basic documents, a systematic review of EtD criteria, input from a range of stakeholders, and a thematic analysis to identify key domains [41].

It was difficult to discern the expertise of contributors to framework development; most groups appeared to consist mainly of academic, generalist guideline methodologists with either clinical or public health experience. Social scientists led work on WHO-INTEGRATE [38] and contributed to the GRADE public health framework [6]. Eight of the 14 organizations reported who funded the development of the EtD framework [2, 11, 21, 24, 27, 34, 37, 38] and five reported declarations of interest among framework developers [2, 11, 20, 24, 38]; the remaining organizations did not provide this information.

#### Decision criteria

**Fig. 1:**
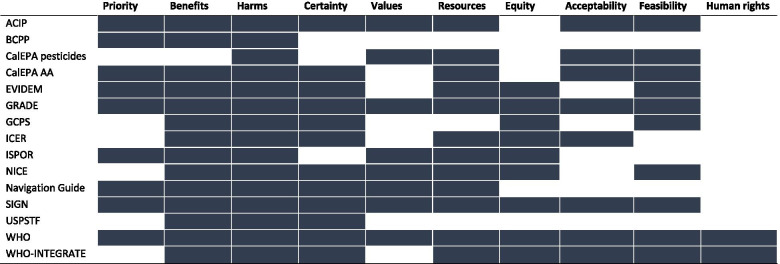
Evidence-to-decision criteria for each key organization. Legend. Across the top of the figure, broad categories of evidence-to-decision criteria are presented. For each listed organization, the cell is shaded if their evidence-to-decision framework encompasses one or more criteria within a category.

Eighteen frameworks were examined in detail: one from each of the 14 organizations, except for CalEPA and GRADE, where two [22, 23] and four [6, 9, 11, 17] unique frameworks were examined, respectively. There were significant commonalities across frameworks in the criteria for formulating recommendations (Table 3 and Fig. 1). Unsurprisingly, all included consideration of benefits, except the CalEPA framework for pesticides [22] which examines risks (of environmental exposures) and not benefits. All frameworks included an assessment of harms of the intervention under consideration. Fifteen of the 18 frameworks included some assessment of certainty or quality of the body of evidence across outcomes in the decision-making process: BCPP, one of the CalEPA frameworks, and ISPOR (International Society for Pharmacoeconomics and Outcomes Research) did not [21, 22, 28]. Some measure of costs, resource use or cost-effectiveness was found in all frameworks except BCPP, USPSTF and the GCPS [21, 26, 36]. Other decision criteria were variably included: feasibility (13 frameworks), equity (12), values (11), and acceptability (11). Only two frameworks included human rights: WHO [37] and WHO-INTEGRATE frameworks [41].

**Table 3:** Criteria included in evidence-to-decision frameworks

		Priority	Benefits and harms				Values		Economc implications		
	Organization	Priority of the problem	Desirable effects	Undesirable effects	Certainty of evidence regarding desirable and undesirable effects	Balance of effects	Values	Certainty of evidence regarding values	Resource considerations	Certainty of evidence regarding resources	Cost-effectiveness
1	**Advisory Committee on Immunization Practices (ACIP) (US Centers for Diseased Control and Prevention)** [20, 39]	Is the problem of public health importance?	How substantial are the desirable anticipated effects?	How substantial are the undesirable anticipated effects?	What is the overall certainty of this evidence for the critical outcomes?	Do the desirable effects outweigh the undesirable effects?	Does the target population feel that the desirable effects are large relative to undesirable effects?	Is there important uncertainty about or variability in how much people value the main outcomes?	Is the intervention a reasonable and efficient allocation of resources?	NI	Is the intervention a reasonable and efficient allocation of resources? (Cost-effectiveness is included in the explanatory text.)
2	**Breast Cancer Prevention Partners (BCPP)** [21]	Does the intervention address cross-cutting, systemic problems?	Is there evidence that the intervention has been successful in the past or does it show potential for success?	Was there general agreement that the intervention would do no harm, i.e. not create unintended consequences?	NI	NI	NI	NI	NI	NI	NI
3	California Environmental Protection Agency (CalEPA) [22]	NI	NI	Deciding whether the proposed or current use of a pesticide results in an unacceptable risk; identifying options to minimize those risks	NI	NI	Evaluating those options according to a value system that includes scientific, social, legal and economic factors, as well as practicality and enforceability.	NI	Evaluating those options according to a value system that includes scientific, social, legal and economic factors, as well as practicality and enforceability.	NI	NI
	California Environmental Protection Agency (CalEPA) [23]	Exposure assessment (identify exposure pathways and estimate the exposure impact, including chemical quantities and household, workplace and market presence across the lifecycle)	Product function (service or utility the product provides) and performance: includes: Principal manufacturer-intended uses or applications; functional and performance attributes, and relative function and performance; applicable legal requirements; useful life of the product; whether an alternative exists that is functionally acceptable, technically feasible, and economically feasible.	Adverse impacts (Adverse environmental impacts; Adverse public health impacts; Adverse waste and end-of-life impacts; Environmental fate Materials and resource consumption impacts; Physical chemical hazards; Physicochemical properties, Associated exposure pathways and life cycle segments).	Uncertainty analysis performed for individual factors assessed (sensitivity analysis or scenario analysis)	NI	NI	NI	Economic impacts (costs) Public health and environmental costs; cost to government agencies and non-profit organizations; internal cost Materials resource and consumption impacts	NI	NI
4	Evidence and Values Impact on DEcision Making (EVIDEM) [24]	Disease severity; size of affected population; unmet needs; population priorities and acess	Comparative effectiveness; comparative patient-perceived health / patient-reported outcomes, type of preventive or therapeutic benefit	Comparative safety/tolerability	Quality of evidence (validity, relevance, completeness of reporting, type of evidence)	NI	NI	NI	Comparative cost consequences - cost of intervention, other medical costs, non-medical costs; Opportunity costs and affordability	NI	NI
5	**Grading of Recommendations, Assessment, Development and Evaluation (GRADE) (clinical - individual perspective)** [17]	Is the problem a priority (from the perspective of an individual patient)?	How substantial are the desirable anticipated effects?	How substantial are the undesirable anticipated effects?	What is the overall certainty of the evidence of effects?	Does the balance between desirable and undesirable effects favour the intervention or the comparison?	Is there important uncertainty about, or variability in, how much people value the main outcomes?	(combined with values criterion)	How large are the resource requirements (costs)?	What is the certainty of the evidence of resource requirements (costs)?	Does the cost-effectiveness of the intervention (the out-of-pocket cost relative to the net desirable effect) favour the intervention or the comparison?
	**GRADE (clinical - population perspective)** [17]	Is the problem a priority?	Same as clinical recommendations, individual perspective	Same as clinical recommendations, individual perspective	Same as clinical recommendations, individual perspective	Same as clinical recommendations, individual perspective	Same as clinical recommendations, individual perspective	Ni	Same as clinical recommendations, individual perspective	Same as clinical recommendations, individual perspective	Does the cost-effectiveness of the intervention favor the intervention or the comparison?
	**GRADE (coverage decisions)** [9]	Is the problem a priority?	Same as clinical recommendations, individual perspective	Same as clinical recommendations, individual perspective	Same as clinical recommendations, individual perspective	Does the balance between desirable and undesirable effects favour the option or the comparison?	Is there important uncertainty about how much people value the main outcomes?	Ni	How large are the resource requirements (costs)?	What is the certainty of the evidence of resource use?	Does cost-effectiveness favor the option or the comparison?
	**GRADE (health system or public health decisions or recommendations)** [6]	Is the problem a priority?	Same as clinical recommendations, individual perspective	Same as clinical recommendations, individual perspective	Same as clinical recommendations, individual perspective	Same as clinical recommendations, individual perspective	Same as clinical recommendations, individual perspective	Ni	Same as clinical recommendations, individual perspective	Same as clinical recommendations, individual perspective	Does the cost-effectiveness of the intervention favor the option or the comparison?
6	**Guide Community Preventive Services (US Centers for Diseased Control and Prevention)** [26]	___	Benefits	Harms	“Strength of evidence” based on number of studies, study design, quality of execution, consistency, and meaningful effect	Not explicit	___	Ni	NI	NI	(Examined but does not contribute to decisions.)
7	**Institute for Clinical and Economic Review (ICER)** [27]	___	Comparative clinical effectiveness (involves weighing the benefits and harms/burdens of one treatment option versus another)	Potential other benefits or disadvantages	Confidence in the body of evidence and the accuracy of estimates of risks and benefits; certainty of net benefit	Comparative clinical effectiveness (involves weighing the benefits and harms/burdens of one treatment option versus another)	___	Ni	Potential budget impact (for short-term affordability assessment)	NI	Incremental cost-effectiveness; long-term value for money
8	**International Society for Pharmacoeconomics and Outcomes Research (ISPOR)** [29, 30]	Severity of disease	Assessed as QALYs	Assessed as QALYs	___	___	Value of hope; Also (paraphrased): value as incorporated into QALYS	Ni	Net costs (resulting directly from the intervention)	NI	QALYs gained
9	**National Institutes for Health and Care Excellence (NICE) (UK)** [32]	__	Benefits	Harms	Quality/certainty of the evidence	Balance of benefits and harms; magnitude and importance of the benefits and harms of an intervention, and the potential for unintended consequences.	Relative values placed on outcomes	Ni	Costs, resource use and economic considerations	NI	Cost effectiveness and other types of economic analysis
10	**Navigation Guide** [2, 16, 40]	Exposure prevalence	Benefits	Assessment of risk of adverse health outcomes from (paraphrased) “exposure to a chemical or class of chemicals or other environmental exposure”	Assess quality of evidence (on risk or toxicity)	__	Values and preferences	Ni	Costs and benefits	NI	NI
11	**Scottish Intercollegiate Guideline Network (SIGN)**^**a**^ [34]	Is this question a priority?	What benefit will the proposed intervention/action have?	What harm might the proposed intervention/action do?	Quality of evidence (Subcriteria: How reliable are the studies in the body of evidence? Are the studies consistent in their conclusions? Are the studies relevant to our target population? Are there concerns about publication bias?)	Balancing benefits and harms	How do patients value different outcomes?	Ni	Is the intervention /action implementable in the Scottish context? Consider existing SMC advice, cost effectiveness, financial, human and other resource implications.	NI	Is the intervention/action implementable in the Scottish context? Consider existing SMC advice, cost effectiveness, financial, human and other resource implications.
12	**US Preventive Services Task Force (USPSTF)** [36]	NI	Benefits	Harms	Certainty of net benefit	Magnitude of net benefits	Ni	Ni	NI	NI	NI
13	**World Health Organization (WHO)** [37]	Priority of the problem	NI	NI	Quality of the evidence	Balance of benefits and harms	Values and preferences	Ni	Resource implications	NI	NI
14	**WHO INTEGRATE** [38, 41])	NI	NI	NI	Quality of evidence	Balance of benefits and harms	NI	Ni	Financial and economic considerations	NI	NI

Other								
Equity	Acceptability	Feasibility	Autonomy	Sustainability	Legal and regulatory considerations	Political considerations	Human rights	Other considerations
NI	Is the intervention acceptable to key stakeholders?	Is the intervention feasible to implement?	NI	NI	NI	NI	NI	Framework includes additional criteria “balance of consequences”
NI	NI	NI	NI	NI	NI	NI	NI	Does the intervention support the science-based intervention goals? Is the intervention in alignment with the Guiding Principles of *Paths to Prevention*? Mentioned in the text only: Can the intervention be implemented in a wide range of realms?
NI	Evaluating those options according to a value system that includes scientific, social, legal and economic factors, as well as practicality and enforceability.	Evaluating those options according to a value system that includes scientific, social, legal and economic factors, as well as practicality and enforceability.	NI	NI	Evaluating those options according to a value system that includes scientific, social, legal and economic factors, as well as practicality and enforceability.	NI	NI	Identifying options to minimize those risks Selecting an effective course of action to reduce or eliminate unacceptable health or environmental risks
NI	“Whether an alternative exists that is functionally acceptable..” - Table 3-1D (page 233)	“Whether an alternative exists that is ... technically feasible…” (page 233)	NI	NI	“to identify the product function, performance, and the legal requirements of the Priority Product and alternatives to ensure the selected choice is feasible”	NI	NI	Life cycle impacts (from raw materials extraction through end-of-life disposal)
(Subcriteria of “population priorites and acess”: special populations, rare diseases, etc.)	NI	Mandate and scope of the health system; System capacity and appropriate use of intervention; Political, historical and cultural context	(Sub-criteria of”comparative patient-perceived health”: impact on autonomy)	NI	NI	NI	NI	Expert consensus/clinical practice guidelines Common goals and specific interests Environmental impact
What would be the impact on health equity?	Is the intervention acceptable to patients, their caregivers, and healthcare professionals?	Is the intervention feasible for patients, their caregivers and healthcare providers?	NI	NI	NI	NI	NI	NI
Same as clinical recommendations, individual perspective	Is the intervention acceptable to key stakeholders?	Is the intervention feasible to implement?	NI	NI	NI	NI	NI	NI
Same as clinical recommendations, individual perspective	Is the option acceptable to stakeholders?	Is the option feasible to implement?	NI	NI	NI	NI	NI	NI
Same as clinical recommendations, individual perspective	Is the option acceptable to key stakeholders?	Is the option feasible to implement?	NI	NI	NI	NI	NI	NI
What would be the impact on health equity?	NI	“Barriers” - a secondary consideration	NI	NI	NI	NI	NI	Evidence gaps Applicability to US populations, US settings of implementation, and intervention characteristics
Degree to which the intervention differentially benefits a historically disadvantaged or underserved community	Delivery mechanism or relative complexity may affect adherence and outcomes	NI	NI	NI	NI	NI	NI	“...Impact of the condition on family and caregivers...” “...Impact on improving return to work/or overall productivity...” “…potential effectiveness of future treatments…” Similarity of mechanism of action to that of other active treatments
Equity	NI	NI	NI	NI	NI	NI	NI	Labor productivity Adherence-improving factors Value of reducing uncertainty due to a new diagnostic Fear of contagion (benefit in reducing anxiety of future disease spread) Insurance value (physical and financial risk protection from new treatments) Value of hope (uncertainty: benefit may be greater than the mean) Real option value (opportunity to benefit from future advances in medicine) Scientific spillover (impact of a new technology on future patients)
Impact on health inequities	NI	Feasibility of implementation	NI	NI	NI	NI	NI	Effect on population health, if appropriate to the topic; any ethical issues, social value judgements, equity considerations and inequalities in outcomes, and policy imperatives, as well as equality legislation
NI	NI	NI	NI	NI	NI	NI	NI	Availability of safer alternatives
Equity	Is the intervention/action acceptable to patients and carers compared to comparison? Consider benefits vs harms, quality of life, other patient preferences.	Is the intervention/action implementable in the Scottish context? Consider existing SMC advice, cost effectiveness, financial, human and other resource implications	NI	NI	NI	NI	NI	“Are there any common comorbidities that could have an impact on the efficacy of the intervention? “(in the “considered judgement pro-forma 2014″ form). “Equity” and “How do patients value the different outcomes?” are mentioned in the SIGN 50 manual but not in the “considered judgement pro-forma 2014″ form.
NI	NI	NI	NI	NI	NI	NI	NI	Other factors can be considered for determing the adequacy of evidence for a recommendation: prevalence or natural history of the target condition, and biological plausibility, clinical relevance and applicability of the evidence, among others. “Grade changes may also result from changes in context (clinical context, societal values for specifc outcomes, and context of intervention and treatment.”.
Equity and human rights	Acceptability	Feasibility	NI	NI	NI	NI	Equity and human rights	NI
Health equity, equality and non-discrimination	Human rights and sociocultural acceptability	Feasibility and health system considerations	NI	NI	NI	NI	Human rights and sociocultural acceptability	Societal implications

(*) The criteria listed here are from both the “Considered judgement pro-forma 2014” form and the headings in the SIGN 50 guideline handbook as these differ.

Abbreviations: *NI* not included

Footnotes

(^a^) The criteria listed here are from both the “Considered judgement pro-forma 2014” form and the headings in the SIGN 50 guideline handbook as these differ.

“Priority of the problem” was explicitly included in the EtD frameworks based on GRADE [20, 34, 37], as well as the BCPP [21], EVIDEM (Evidence and Values Impact on DEcision Making) [24], ISPOR [28–30] and Navigation Guide frameworks [16]. However, in the background materials for several other frameworks, the burden of the disease was mentioned as an important consideration when prioritizing an intervention for development of a guideline or policy.

The meaning of the criterion “values and preferences” varied over time and across publications. This phrase encompasses two different constructs: the relative value that persons affected by the recommendations place on the outcomes of the intervention and the preferences such persons have regarding the intervention options. The GRADE Working Group initially combined these two constructs [5] with a definition encompassing both [43]. The 2016 update of the GRADE EtD framework introduced two separate criteria: variability of the value affected persons place on the main outcomes, and acceptability of the intervention [11] to various stakeholders. Other key organizations include a decision criterion related to values using verbiage closely aligned with GRADE [16] and focused exclusively on the relative value of outcomes [32, 34], or they include the concept but with a somewhat different meaning [20, 22, 28].

#### Evidence used to inform each criterion when organizations make decisions

All of the frameworks report that the EtD criteria should be informed by evidence obtained from a variety of sources, with an emphasis on systematic reviews of research evidence (Table 2). Twelve of the organizations recommend an assessment of the quality (validity or certainty) of the body of evidence for important outcomes as part of the evidence review, and most recommend the GRADE system with or without modifications. Only BCPP [21] and one of the CalEPA guidelines [22], do not recommend such an assessment. The approaches used by the GCPS [26] and USPSTF [36] differ somewhat from GRADE in their assessment of the “strength of evidence” and “certainty of net benefit”, respectively. The EtD frameworks with an economic focus describe varied approaches to assessing quality of the body of evidence [24, 27, 28].

#### Nomenclature for recommendations

Nine of the included organizations provide specific guidance on the nomenclature for the types of recommendations formulated based on the EtD criteria (Table 2). The most common categorization of recommendations among those nine organizations was two levels of strength both for and against a recommendation, i.e., four categories [11, 34, 37]. GRADE has extensive documents on this issue and uses the terms “strong” and “weak or conditional “[5]. The USPSTF has five categories of recommendations [34], while ACIP [37] and the GCPS [35] have three. The economic-focused frameworks refer to “value” in various ways [24, 27, 30]. NICE uses the wording of recommendations to reflect the strength of the evidence (e.g., offer, advise, consider) rather than standardized terms to represent the strength of the recommendation [32].

Five organizations provide explicit guidance on the situations where recommendations cannot be formulated due to insufficient evidence or a close balance between benefits and harms (Table 2) [11, 20, 26, 32, 36]. The USPSTF provides the most detailed guidance on this situation [44]. Ten of the organizations suggest including knowledge or research gaps with the recommendations, with particular emphasis in ACIP, NICE, GCPS and the USPSTF.

#### Specific evidence-to-decision frameworks

GRADE includes EtD frameworks for four different purposes: i) clinical recommendations, individual perspective; ii) clinical recommendations, population perspective; iii) coverage decisions; and iv) health system and public health recommendations/decisions. (There is a fifth GRADE EtD framework for diagnostic, screening and other tests but this was not included in this review of interventions considered most relevant for application to environmental health [11].) These frameworks are all very similar, all with 12 criteria covering the same concepts, with some variation in verbiage, tailored to the different audiences (Table 3). There is more emphasis on resource considerations, equity, acceptability and feasibility for health systems and public health decisions than for individual patient clinical recommendations [6, 9].

ACIP’s EtD framework is derived directly from GRADE [20], while NICE [31] and SIGN’s [34] EtD criteria closely resemble those of GRADE. WHO uses the GRADE EtD framework which was current when their guidance was published in 2014 [37].

The WHO-INTEGRATE EtD framework version 1.0 [38, 41], first published in 2019 (Table 2), was developed in response to a perceived need to take a complexity perspective into account, to develop a conceptual framework to underpin the EtD, and to incorporate public health and WHO-specific values when developing WHO guidelines. WHO-INTEGRATE includes six broad criteria [38] (Table 3), with quality of evidence applied to all criteria. Each criterion includes sub-criteria: for example, the main criterion “Health equity, equality and non-discrimination” [38] includes impact on health equality and/or health equity, distribution of benefits and harms, affordability, and accessibility.

The frameworks that focus on interventions in environmental health were generally less well developed and lacked specificity compared with the clinical and public health frameworks [2, 21, 22] with the exception of a recently published guide to alternatives analysis for chemicals in consumer products [23].

Additional details on each of the included frameworks are provided in Additional file 1.

## Discussion

A number of EtD frameworks have been developed in a variety of clinical, and public and environmental health disciplines, and there is significant overlap in the criteria used to inform recommendations or decisions across these frameworks. Benefits and harms are almost universally included, and an assessment of certainty or quality of the body of evidence and some measure of resource use were included in most frameworks examined. Other decision criteria such as values, equity, feasibility, and acceptability were variably included, while only two frameworks encompassed human rights. There was variation across frameworks in terminology, definitions and presentation of EtD criteria. The five systematic reviews of EtD frameworks describe decision criteria that were similar to those presented in the frameworks from key organizations.

The 18 frameworks identified for 14 key organizations focused mainly on clinical medicine or public health interventions. The GRADE framework is the most well developed, with extensive information and guidance on its methods. A number of the key organizations examined have adopted GRADE, often with minor modifications [16, 20, 32, 34, 37]. Three of the four frameworks related to environmental health lacked detail on the specific criteria for decision-making [2, 21, 22]. Frameworks originating in the HTA realm unsurprisingly focus on economic considerations [24, 27, 28], while also including criteria similar to those encompassed by other frameworks.

### General aspects of evidence-to-decision frameworks

The process and methods for developing EtD frameworks were often superficial and poorly reported. Only WHO-INTEGRATE [41] and the GRADE coverage [8] frameworks were developed following a systematic review of existing frameworks or potential decision criteria. Only WHO-INTEGRATE describes a conceptual framework [38] underpinning the EtD criteria. The lack of a conceptual framework represents a significant weakness in most frameworks: key considerations may be missed and long-standing criteria may be perpetuated without adequate scrutiny.

GRADE has dominated guideline methods in health care and public health over the last 15 years. This has led to productive collaborations, standardization of processes and methods, and the development of detailed methods and guidance. On the other hand, there are instances where intellectual dominance by a single group – i.e., monopolies of knowledge – can suppress innovation and slow down development processes [45].

EtD frameworks largely focus on, and are optimal for, single-component interventions with simple linear pathways from intervention to outcomes. In addition, most EtD frameworks do not consider the context in which the intervention is delivered. With the exception of WHO-INTEGRATE, EtD frameworks do not explicitly or even implicitly incorporate a complexity perspective including the inter-relationship between the intervention and the context or environment in which it is delivered. Only the GCPS, the USPSTF, and WHO-INTEGRATE recommend the use of visual depictions of the relationships among the intervention components: such approaches facilitate examination of multiple key questions linked across the causal pathway for intervention effects, as well as the consideration of contextual factors which may be important for decision making.

Guidance on if and when to identify, synthesize and integrate evidence on criteria other than benefits and harms of the intervention into the decision-making process was provided only for WHO-INTEGRATE [38]. In our experience, most guideline development processes focus almost exclusively on benefits and harms in the evidence review and in discussions. Recently, however, guideline groups are examining qualitative evidence on acceptability, feasibility, and other decision criteria. Guideline groups continue to be uncertain as to how to incorporate EtD criteria such as equity, into recommendations in a meaningful fashion.

Ideally, EtD considerations are discussed early in the guideline development process, and strategic decisions are made as to the types of information and evidence that will be needed to inform key considerations. It may not be necessary, feasible, or even possible, to systematically examine evidence on all EtD considerations. The participation of a range of stakeholders, including persons and communities potentially affected by the intervention, is important both early in the planning phase and later when decisions are made. Their perspectives and experiences with issues such as acceptability and feasibility, for example, are critical for formulating credible and impactful recommendations or policies.

### Evidence-to-decision criteria across frameworks

It was difficult to compare specific EtD criteria across frameworks due to significant variation in approaches to lumping and splitting criteria across frameworks, as well as in the terminology and definitions used. While most frameworks presented broad categories of criteria (e.g., “equity”), only WHO-INTEGRATE [38] provides detailed sub-criteria to facilitate understanding and decision making. Some main criteria included several constructs, which can complicate the decision-making process and reporting of the rationale for the recommendation or decision. For example, WHO-INTEGRATE’s “Human rights and sociocultural acceptability” includes both human rights and what other frameworks refer to as “preferences” or “acceptability”.

There is also variability in how criteria are defined and whether operational definitions or guidance are provided. The criterion “values and preferences” is particularly problematic and guideline developers continue to use the phrase “values and preferences” with unclear and variable meaning.

The GRADE criterion of “priority of the problem” is also problematic. It was initially included in the GRADE framework to facilitate prioritization across interventions, such as at the national or sub-national levels. However, in most guideline development scenarios, the problem on which interventions and comparators are focused has already been determined to be of high priority. Thus, examination of the burden of disease and other priority considerations is irrelevant at the stage of recommendation formulation.

The criteria related to resource use vary considerably, and may include cost, affordability, infrastructure needs, personnel training, and measures of economic efficiency (e.g., cost-benefit and cost-effectiveness). This variability is due in part to the different perspectives of the sponsors of the EtD framework or of the target audiences for the organization’s products. For example, the USPSTF is prohibited by national legislation from considering cost or cost-effectiveness in their recommendations. On the other hand, for the UK’s NICE, cost-effectiveness is a critical part of their decisions. There is also variability in the extent and specificity of the guidance on how to incorporate resource use into decision-making. While well-developed for NICE [32], and reasonably so for GRADE [46], guidance is almost completely lacking for WHO [37] and WHO-INTEGRATE [38].

The criterion of “equity” is poorly described and little operational guidance is provided across the frameworks except for WHO-INTEGRATE which provides several sub-criteria for consideration under the main criterion of “health equity, equality and non-discrimination” [38].

### Group decision making

Group decision making when formulating recommendations in guidelines is rarely a simple, linear process. While EtD frameworks support the normative aspect of decision making in terms of how expert panels “should” or “ought to” develop recommendations by providing a structure for decision making [47–49], many factors affect how groups make decisions [47]. These include: i) situational or contextual factors (e.g., time pressure, social context, gender bias, political pressures); ii) individual characteristics of the decision maker (e.g., role on the expert panels, cultural and professional background, race/ethnicity, methodological expertise) [50]; and iii) individual panel member’s emotions and experiences (e.g., personal experiences with a disease). How these various factors contribute to decision making and recommendation formulation in guidelines is unknown [47], but their potential effects must be kept in mind and made explicit to the extent possible.

While acknowledging the complex process of formulating recommendations in guidelines, EtD frameworks are a valuable tool and have led to vast improvements over “free-for-all” meetings where decision making criteria were selected in an ad hoc manner, and it was often unclear which considerations actually contributed to final decisions and their relative weight, what evidence was examined, and how and why the final recommendation or decision was arrived at. EtD frameworks can be combined with any one of a number of approaches for achieving group consensus on the direction, strength, and wording of a recommendation or decision, including formal approaches such as the Delphi approach and the Nominal Group Technique, or voting if consensus cannot be reached.

### Applying clinical and public health frameworks to environmental health

Applying approaches for clinical medicine interventions to other scientific fields, including environmental health, is challenging. There are important differences both in the assessment of the quality (certainty) of the body of evidence, and in translating evidence to recommendations or decisions. Woodruff and colleagues [16] note that the GRADE system and other evidence-based medicine approaches have limitations in terms of applicability to questions and decision making in environmental health. The reasons include: i) the need to combine human, animal and (sometimes) mechanistic evidence; ii) the paucity of randomized controlled trials (RCTs) and other types of experimental studies in humans due to ethical considerations; and iii) differences in the decision-making context (e.g., weighing the benefits and harms is different for clinical interventions than for unintended exposures to substances in the environment) [16]. Nonetheless, the constructs of the GRADE and WHO-INTEGRATE frameworks likely apply to environmental health interventions.

### Limitations of this analysis

The approach taken for this review and analysis has a number of limitations. The methods for identifying existing frameworks were limited: the systematic review included only reviews and focused only on English language literature accessible through PubMed. Furthermore, many EtD frameworks are not published in the peer reviewed literature: rather, they are found on organizational web-sites and/or in organizational procedure manuals. For the review of key organizations, only a convenience sample was examined and EtD frameworks with important additional considerations or novel approaches may have been overlooked. However, it is unlikely that this approach missed key or significantly different frameworks in view of the dominance of GRADE and our consultation with experts on EtD frameworks.

Our analysis also has limitations related to the nature of the available data. Published information on the methods for developing frameworks and for operationalizing them was generally sparse, except for GRADE and WHO-INTEGRATE. For some frameworks it was difficult to identify exactly which criteria were routinely included in the decision-making process. While the publication might present a list or table of criteria, additional criteria might be mentioned in the adjacent text. However, the degree to which the guideline group or other decision maker addressed these additional criteria was often unclear.

## Conclusions and next steps

EtD frameworks are an extremely useful tool for recommendation formulation and decision making in healthcare and other scientific fields. They enable decisions to be made based on research evidence and with explicit consideration of a range of constructs, and they facilitate clear articulation of the rationale for decisions.

The principles underpinning evidence-informed, transparent and impactful decision making are the same across scientific fields, including environmental health. The GRADE EtD framework, along with WHO-INTEGRATE with its focus on contextual issues and inter-relationships, provide a useful starting point for consideration for decision-making for interventions in environmental health. Significant modifications will be needed, however, given the nature of the evidence base and the complex context in which environmental health interventions are designed, implemented, regulated and evaluated. The process for developing an EtD framework for environmental health interventions requires a broad range of experts, including not only environmental health scientists, but evidence synthesis and guideline methodologists, public health generalists, human rights and ethics experts, social scientists, and economists, among others. An iterative development process will be needed: a draft framework should be pilot-tested, revised and evaluated. The framework’s utility and impact on decision making, and the quality and impact of the resultant recommendations need careful evaluation, with the results used to develop future iterations.

### Supplementary Information

**Additional file 1.**

## Data Availability

All data generated or analysed during this study are included in this published article.

## Appendix

### Annex 1. Search strategy and inclusion criteria

((“Policy Making”[Mesh]) OR (“Decision Making”[Mesh])) AND (((framework[Title/Abstract]) OR (frameworks[Title/Abstract])) OR ((template[Title/Abstract]) OR (templates[Title/Abstract]) OR (tool[Title/Abstract]) OR (tools[Title/Abstract]))).

Filters: English language, systematic review, last 10 years.

The search was executed 7 January 2021 in the PubMed search engine for Medline.

### Annex 3. Results of the search: full-text review

The search of PubMed performed on 7 Jan 2021. 399 citations were identified, 25 of which were reviewed in full text: these are listed below with the reason for exclusion.

Abbreviations: E, excluded citation; E2D, evidence-to-decision; I, included citation; LMICs, low- and middle-income countries; NA (not applicable because the publication fulfilled inclusion criteria)

**Table:**

Citation	Title	Include/exclude	Rationale for exclusion
Baltussen 2019 [1]	Multicriteria decision analysis to support health technology assessment agencies: Benefits, limitations, and the way forward	I	NA
Boivin 2018 [2]	Patient and public engagement in research and health system decision making: A systematic review of evaluation tools	E	Review of tools for evaluating patient and public engagement, not for decision making
Burchett 2012 [3]	National decision-making on adopting new vaccines: a systematic review	I	NA
Carroll 2013 [4]	Best fit framework synthesis: refining the method	E	Describes “best fit” method for qualitative synthesis; no EtD frameworks
Carvalho 2018 [5]	Capturing budget impact considerations within economic evaluations: A systematic review of economic evaluations of rotavirus vaccine in low- and middle-income countries and a proposed assessment framework	E	Checklist for budget impact analyses
Clark 2014 [6]	Discrete choice experiments in health economics: A review of the literature	E	Review of discrete chose experiments; no examination of EtD frameworks
Clark 2018 [7]	Measuring trade-offs in nephrology: A systematic review of discrete choice experiments and conjoint analysis studies	E	Review of discrete choice experiments in nephrology
Clarke 2016 [8]	The application of theories of the policy process to obesity prevention: a systematic review and meta-synthesis	E	Review of studies where theory underpins the policy development process or adoption of policies. Focus is on explaining the policies and not on what factors should be considered in decision-making.
Clifford 2017 [9]	What information is used in treatment decision aids? A systematic review of the types of evidence populating health decision aids	E	Focus on patient decision aids
Dodd 2019 [10]	Investigating the process of evidence-informed health policymaking in Bangladesh: a systematic review	E	Reviews factors linked to policy uptake or prioritization and how and why evidence is used; presents explanatory and descriptive information and not frameworks for normative decisions
Gentil 2015 [11]	A systematic review of socio-economic assessments in support of coastal zone management (1992–2011)	E	Describes concepts used for decision-making; presents descriptive information and not normative frameworks
Kolasa 2015 [12]	Pricing and reimbursement frameworks in Central Eastern Europe: a decision tool to support choices	E	Review revealed 33 pricing and reimbursement schemes; led to framework with two economic questions. Exclude as exclusively economic focus.
Kristensen 2019 [13]	Identifying the need for good practices in health technology assessment: Summary of the ISPOR HTA Council Working Group Report on Good Practices in HTA	E	Neither this paper or the full report (https://www.ispor.org/member-groups/councils-roundtables/health-technology-assessment-council) provides a review of EtD frameworks (referred to as “contextualization”).
Lam 2018 [14]	Decision support tools for regenerative medicine: Systematic review	E	Examines decision support tools for product development and manufacturing of cell and gene therapies in regenerative medicine; does not focus on clinical medicine, public health or environmental health
LaRocca 2012 [15]	The effectiveness of knowledge translation strategies used in public health: A systematic review	E	Review of evaluations of knowledge translation strategies and not the strategies themselves
Laurans 2013 [16]	Use of ecosystem services economic valuation for decision making: Questioning a literature blindspot	E	Focuses on the types and uses of Ecosystem Services economic Valuation (ESV), not on the criteria for decision making
Liverani 2013 [17]	Political and institutional influences on the use of evidence in public health policy. A systematic review	E	Examines factors that impact the use of evidence in policy-making
Mayer 2017 [18]	Costing evidence for health care decision making in Austria: A systematic review	E	Examines economic analyses and costing methods used in Austria; does not present EtD frameworks
Mustafa 2017 [19]	Decision making about healthcare-related tests and diagnostic test strategies. Paper 3: a systematic review shows limitations in most tools designed to assess quality and develop recommendations	I	NA
Sullivan 2015 [42]	What guidance are economists given on how to present economic evaluations for policymakers? A systematic review	E	Review of reporting guidance for economic analyses; does not deal directly with EtD concepts
Trapero-Bertram 2019 [33]	What attributes should be included in a discrete choice experiment related to health technologies? A systematic literature review	E	Review of attributes for discrete choice experiments; not a review of EtD frameworks
Tsoi 2013 [35]	Harmonization of reimbursement and regulatory approval processes: a systematic review of international experiences	E	Review of approaches to harmonize reimbursement and regulatory approval processes
Tsoi 2015 [39]	Systematic narrative review of decision frameworks to select the appropriate modelling approaches for health economic evaluations	E	Examines frameworks for selecting economic modelling approaches
Votruba 2018 [25]	A systematic review of frameworks for the interrelationships of mental health evidence and policy in low- and middle-income countries	E	Review of theories and frameworks explaining use of evidence to formulate policies in mental health in LMICs; does not review frameworks for decision-making
Wickremasinghe 2016 [31]	District decision-making for health in low-income settings: A systematic literature review	I	NA
**From other sources/ad hoc**			
Domrumel 2020 [37]	Organizational aspect in healthcare decision-making: a literature review	E	Systematic review of individual criteria for decision-making (not a review of frameworks or sets of criteria)
Morgan 2018 [38]	Decision-making frameworks and considerations for informing coverage decisions for healthcare interventions: a critical interpretive synthesis.	I	NA

#### References for the full-text review

1. Baltussen R, Marsh K, Thokala P, et al. Multicriteria Decision Analysis to Support Health Technology Assessment Agencies: Benefits, Limitations, and the Way Forward. *Value Health.* 2019;22(11):1283–1288.

2. Boivin A, L’Espérance A, Gauvin FP, et al. Patient and public engagement in research and health system decision making: A systematic review of evaluation tools. *Health Expect.* 2018;21(6):1075–1084.

3. Burchett HE, Mounier-Jack S, Griffiths UK, Mills AJ. National decision-making on adopting new vaccines: a systematic review. *Health Policy Plan.* 2012;27 Suppl 2:ii62–76.

4. Carroll C, Booth A, Leaviss J, Rick J. “Best fit” framework synthesis: refining the method. *BMC Med Res Methodol.* 2013;13:37.

5. Carvalho N, Jit M, Cox S, Yoong J, Hutubessy RCW. Capturing Budget Impact Considerations Within Economic Evaluations: A Systematic Review of Economic Evaluations of Rotavirus Vaccine in Low- and Middle-Income Countries and a Proposed Assessment Framework. *Pharmacoeconomics.* 2018;36(1):79–90.

6. Clark MD, Determann D, Petrou S, Moro D, de Bekker-Grob EW. Discrete choice experiments in health economics: a review of the literature. *Pharmacoeconomics.* 2014;32(9):883–902.

7. Clark MD, Szczepura A, Gumber A, Howard K, Moro D, Morton RL. Measuring trade-offs in nephrology: a systematic review of discrete choice experiments and conjoint analysis studies. *Nephrol Dial Transplant.* 2018;33(2):348–355.

8. Clarke B, Swinburn B, Sacks G. The application of theories of the policy process to obesity prevention: a systematic review and meta-synthesis. *BMC Public Health.* 2016;16(1):1084.

9. Clifford AM, Ryan J, Walsh C, McCurtin A. What information is used in treatment decision aids? A systematic review of the types of evidence populating health decision aids. *BMC Med Inform Decis Mak.* 2017;17(1):22.

10. Dodd M, Ivers R, Zwi AB, Rahman A, Jagnoor J. Investigating the process of evidence-informed health policymaking in Bangladesh: a systematic review. *Health Policy Plan.* 2019;34(6):469–478.

11. Le Gentil E, Mongruel R. A systematic review of socio-economic assessments in support of coastal zone management (1992–2011). *J Environ Manage.* 2015;149:85–96.

12. Kolasa K, Kalo Z, Hornby E. Pricing and reimbursement frameworks in Central Eastern Europe: a decision tool to support choices. *Expert Rev Pharmacoecon Outcomes Res.* 2015;15(1):145–155.

13. Kristensen FB, Husereau D, Huić M, et al. Identifying the Need for Good Practices in Health Technology Assessment: Summary of the ISPOR HTA Council Working Group Report on Good Practices in HTA. *Value Health.* 2019;22(1):13–20.

14. Lam C, Meinert E, Alturkistani A, et al. Decision Support Tools for Regenerative Medicine: Systematic Review. *J Med Internet Res.* 2018;20(12):e12448.

15. LaRocca R, Yost J, Dobbins M, Ciliska D, Butt M. The effectiveness of knowledge translation strategies used in public health: a systematic review. *BMC Public Health.* 2012;12:751.

16. Laurans Y, Rankovic A, Billé R, Pirard R, Mermet L. Use of ecosystem services economic valuation for decision making: questioning a literature blindspot. *J Environ Manage.* 2013;119:208–219.

17. Liverani M, Hawkins B, Parkhurst JO. Political and institutional influences on the use of evidence in public health policy. A systematic review. *PLoS One.* 2013;8(10):e77404.

18. Mayer S, Kiss N, Łaszewska A, Simon J. Costing evidence for health care decision-making in Austria: A systematic review. *PLoS One.* 2017;12(8):e0183116.

19. Mustafa RA, Wiercioch W, Falavigna M, et al. Decision making about healthcare-related tests and diagnostic test strategies. Paper 3: a systematic review shows limitations in most tools designed to assess quality and develop recommendations. *J Clin Epi.* 2017;92:29–37.

20. Sullivan SM, Wells G, Coyle D. What Guidance are Economists Given on How to Present Economic Evaluations for Policymakers? A Systematic Review. *Value Health.* 2015;18(6):915–924.

21. Trapero-Bertran M, Rodríguez-Martín B, López-Bastida J. What attributes should be included in a discrete choice experiment related to health technologies? A systematic literature review. *PloS one.* 2019;14(7):e0219905.

22. Tsoi B, Masucci L, Campbell K, Drummond M, O’Reilly D, Goeree R. Harmonization of reimbursement and regulatory approval processes: a systematic review of international experiences. *Expert Rev Pharmacoecon Outcomes Res.* 2013;13(4):497–511.

23. Tsoi B, O’Reilly D, Jegathisawaran J, Tarride JE, Blackhouse G, Goeree R. Systematic narrative review of decision frameworks to select the appropriate modelling approaches for health economic evaluations. *BMC Res Notes.* 2015;8:244.

24. Votruba N, Ziemann A, Grant J, Thornicroft G. A systematic review of frameworks for the interrelationships of mental health evidence and policy in low- and middle-income countries. *Health Res Policy Syst.* 2018;16(1):85.

25. Wickremasinghe D, Hashmi IE, Schellenberg J, Avan BI. District decision-making for health in low-income settings: a systematic literature review. *Health Policy and Planning.* 2016;31(suppl_2):ii12-ii24.

26. Dubromel A, Duvinage-Vonesch MA, Geffroy L, Dussart C. Organizational aspect in healthcare decision-making: a literature review. *J Mark Access Health Policy.* 2020;8(1):1810905.

27. Morgan RL, Kelley L, Guyatt GH, Johnson A, Lavis JN. Decision-making frameworks and considerations for informing coverage decisions for healthcare interventions: a critical interpretive synthesis. *J Clin Epi.* 2018;94:143–150.

## Abbreviations

AA: Alternatives analysis
ACIP: Advisory committee on immunization practices
BCPP: Breast cancer prevention partners
CalEPA: California environmental protection agency
EVIDEM: Evidence and values impact on decision making
GRADE: Grading of recommendations, assessment, development and evaluation
GCPS: Guide community preventive services
ICER: Institute for clinical and economic review
ISPOR: International society for pharmacoeconomics and outcomes research
NICE: National institutes for health and care excellence
WHO: World health organization
