# Supplementary material for: Evidence-to-decision frameworks: a review and analysis to inform decision-making for environmental health interventions
Source: Environ Health. 2021 Dec 8;20:124. doi: 10.1186/s12940-021-00794-z (PMC8653547; doi:10.1186/s12940-021-00794-z)
Supplement: Supplementary file 1 — Additional file 1. [file 12940_2021_794_MOESM1_ESM.docx]

**Annex 4. Additional details on included evidence-to-decision frameworks**

1. *GRADE evidence-to-decision frameworks*

GRADE includes evidence-to-decision (EtD) frameworks for four different purposes: i) clinical recommendations, individual perspective; ii) clinical recommendations, population perspective; iii) coverage decisions; and iv) health system and public health recommendations/decisions. (There is a fifth GRADE EtD framework for diagnostic, screening and other tests but this was not included in this review of interventions considered most relevant for application to environmental health.^1^) These frameworks are all very similar, all with 12 criteria covering the same concepts, with some variation in verbiage, tailored to the different audiences (Table 3). There is more emphasis on resource considerations, equity, acceptability and feasibility for health systems and public health decisions than for individual patient clinical recommendations.^2,3^

1. *Evidence-to-decision frameworks based directly on GRADE*

ACIP’s EtD framework is derived directly from GRADE,^4^ while NICE^5^ and SIGN’s^6^ EtD criteria closely resemble those of GRADE. WHO uses the GRADE EtD framework which was current when the *WHO Handbook for Guideline Development*, 2^nd^ edition was published in 2014.^7^ Guideline development groups supported by WHO can modify this EtD framework to meet the needs of individual guidelines, and are encouraged to use the current GRADE EtD framework published in 2016.^1,8^

1. *Other evidence-to-decision frameworks*

The WHO-INTEGRATE EtD framework version 1.0,^9,10^ first published in 2019 (Table 2), was developed in response to a perceived need to take a complexity perspective into account and to incorporate public health and WHO-specific values when developing WHO guidelines. The developers also sought to address several weaknesses that they noted in the GRADE EtD:^9^ the absence of a theoretical framework; failure to adequately consider the role of social and economic determinants of health; inadequate consideration of complex interventions, system changes, and the environment in which interventions are implemented; and an undue focus on benefits and harms and not on other considerations critical for decision-making in public health.

WHO-INTEGRATE includes six broad criteria (Table 3): balance of benefits and harms; human rights and sociocultural acceptability; health equity, equality and non-discrimination; societal implications; financial and economic considerations; and feasibility and health system considerations.^9^ A seventh criterion, quality of evidence, is a meta-criterion that applies to each of the six other criteria. WHO-INTEGRATE also includes a number of sub-criteria. For example, the main criterion “Health equity, equality and non-discrimination”^9^ includes several sub-criteria related to the intervention: impact on health equality and/or health equity, distribution of benefits and harms, affordability, and accessibility.

One of the key concepts of the WHO-INTEGRATE approach is the careful consideration of the relative importance of the various EtD criteria at the planning stage of guideline development. Logic models or conceptual frameworks are strongly recommended in order to understand the interrelationships between the intervention and the context in which it is delivered, with prioritization of EtD considerations for the specific decision or recommendation. Relevant evidence is then sought on this subset of criteria.

The US Preventive Services Task Force (USPSTF)^11^ develops an analytic framework depicting the causal pathway by which the intervention may achieve its effects (benefits and harms) and the various questions which must be researched to address the overarching question of net benefit of the intervention. The USPSTF’s recommendations are based primarily on net benefits and the certainty thereof, although other factors may be considered (Table 3).

The US Guide to Community Preventive Services (GCPS) modeled their original processes and methods on those of the USPSTF^12^ with some later modifications (personal communication Dr David Hopkins, 25 January 2021). Based on systematic reviews focused mainly on benefits and harms of the intervention, the beneficial effects of the intervention are assessed as strong, sufficient or insufficient strength of evidence. This assessment is then “upgraded” or “downgraded” based on factors including harms, equity considerations, magnitude of effect and applicability of the evidence to US populations.

Three of the frameworks from key organizations reflect an economic perspective for decision-making in health systems or in decisions on coverage at a system or national level: EVIDEM (Evidence and Values Impact on DEcision Making),^13^ ISPOR International Society for Pharmacoeconomics and Outcomes Research),^14^and ICER (Institute for Clinical and Economic Review).^15^ These frameworks all included consideration of comparative effectiveness. ISPOR presents data as quality adjusted life years (QALYs) and net costs, and includes other considerations such as “value of hope” and “insurance value” (Table 3).^14^ ICER^15^ focuses on comparative clinical effectiveness and incremental cost-effectiveness along with “contextual considerations”, such as “improving return to work and/or overall productivity”. EVIDEM^13^ also focuses on comparative effectiveness, along with cost consequences and other considerations (e.g., “environmental impact”).

1. *Evidence-to-decision frameworks in environmental health*

Four frameworks focused on interventions in environmental health. ^16-19^ These were generally less well developed and lacked specificity compared with the clinical and public health frameworks, except for a recently published guide to alternatives analysis for chemicals in consumer products.^19^ Breast Cancer Prevention Partners (BCPP) developed a comprehensive breast cancer primary prevention plan for the US State of California, focusing on primary prevention and systemic interventions to reduce the incidence of breast cancer, based on the principles of social justice and equity.^16^ This organization prioritized a large number of potential interventions using criteria focused on alignment with the organization’s goals and guiding principles, whether the intervention addresses cross-cutting or systemic problems, past success of the intervention, and potential harms.

California Environmental Protection Agency’s (CalEPA) [Office of Environmental Health Hazard Assessment](http://www.oehha.ca.gov/) (OEHHA) leads California’s assessment of health risks posed by environmental contaminants^20^ and works with the State Department of Pesticide Regulation (DRP) to develop regulations related to pesticide worker health and safety. In response to a review by the US National Academy of Sciences (NAS),^21^ CalEPA published an update of their methods for risk assessment and risk management of pesticides^18^ which focuses on an assessment of a range of options and their risks, and examination of scientific, social, legal, and economic factors, as well as practicality and enforceability.

CalEPA’s Department of Toxic Substances Control Safer Products and Workplaces Program has published a formal approach to alternative analysis of potentially hazardous components of consumer products.^19^ This approach guides decisions on product removal, redesign or other change to reduce the potential impact of the chemical of concern. The analysis compares alternative products across factors including potential chemical exposures, public and environmental health impact, product function and requirements, and economic implications. This analysis considers all relevant life cycle consequences of the chemical or product of concern, from inputs into manufacturing, through end-of-life disposal. While not referenced in the summary “List of Factors for Consideration in Alternatives Analysis” (Appendix 3-1),^19^ other considerations are mentioned in the document text. For example, the entity performing the analysis determines which impacts are most significant based on its values. Likewise, the variable impacts across sensitive subpopulations are to be considered with respect to exposure to or use of the product. Feasibility is also discussed in the text, with respect to the assessment of product function, performance, and the legal requirements.^19^ Finally, acceptability is considered in regards to the function of alternative compounds.

The Program on Reproductive Health and the Environment at the University of California at San Francisco has developed a process and methods for integrating scientific findings from human and nonhuman studies to determine the overall strength of the evidence on the effects of environmental exposures on outcomes in humans.^22^ This approach, published as *The Navigation Guide*^17,22,23^ has adapted the GRADE approach to integrate multiple streams of evidence and a range of study designs, to draw conclusions on the quality and strength of the body of evidence of a substance’s toxicity. This assessment of toxicity is then combined with information on exposure (level, duration and timing) to provide an assessment of risk of adverse health outcomes. This risk assessment could then be combined with consideration of: 1) whether a less toxic agent is available as an alternative; and 2) “values and preferences”, to arrive at a recommendation.^17,23^

**References**

1. Alonso-Coello P, Schünemann HJ, Moberg J, et al. GRADE Evidence to Decision (EtD) frameworks: a systematic and transparent approach to making well informed healthcare choices. 1: Introduction. *BMJ.* 2016;353:i2016.

2. Moberg J, Oxman AD, Rosenbaum S, et al. The GRADE Evidence to Decision (EtD) framework for health system and public health decisions. *Health Research Policy and Systems.* 2018;16(1):45.

3. Parmelli E, Amato L, Oxman AD, et al. GRADE Evidence to Decision (EtD) framework for coverage decisions. *International Journal of Technology Assessment in Health Care.* 2017;33(2):176-182.

4. Lee G, Carr W, Group AE-BRW, et al. Updated framework for development of evidence-based recommendations by the Advisory Committee on Immunization Practices. *Morbidity and Mortality Weekly Report.* 2018;67(45):1271.

5. National Institute for Health and Care Excellence. NICE: The National Institute for Health and Care Excellence. National Institute for Health and Care Excellence. <https://www.nice.org.uk/>. Accessed 1 August, 2020.

6. Scottish Intercollegiate Guidelines Network. *A guideline developer’s handbook.* Edinburgh: SIGN; 2019.

7. World Health Organization. *WHO handbook for guideline development.* 2 ed. Geneva, Switzerland: World Health Organization; 2014.

8. Alonso-Coello P, Oxman AD, Moberg J, et al. GRADE Evidence to Decision (EtD) frameworks: a systematic and transparent approach to making well informed healthcare choices. 2: Clinical practice guidelines. *BMJ.* 2016;353:i2089.

9. Rehfuess EA, Stratil JM, Scheel IB, Portela A, Norris SL, Baltussen R. The WHO-INTEGRATE evidence to decision framework version 1.0: integrating WHO norms and values and a complexity perspective. *BMJ Global Health.* 2019;4(Suppl 1).

10. Stratil JM, Baltussen R, Scheel I, Nacken A, Rehfuess EA. Development of the WHO-INTEGRATE evidence-to-decision framework: an overview of systematic reviews of decision criteria for health decision-making. *Cost Effectiveness and Resource Allocation.* 2020;18(1):8.

11. US Preventive Services Task Force. Home. US Preventive Services Task Force. <https://www.uspreventiveservicestaskforce.org/uspstf/>. Accessed 1 Aug, 2020.

12. Briss PA, Zaza S, Pappaioanou M, et al. Developing an evidence-based Guide to Community Preventive Services—methods. *American Journal of Preventive Medicine.* 2000;18(1):35-43.

13. Goetghebeur MM, Cellier MS. Can reflective multicriteria be the new paradigm for healthcare decision-making? The EVIDEM journey. *Cost Effectiveness and Resource Allocation.* 2018;16(1):54.

14. Lakdawalla DN, Doshi JA, Garrison LP, Jr., Phelps CE, Basu A, Danzon PM. Defining Elements of Value in Health Care-A Health Economics Approach: An ISPOR Special Task Force Report [3]. *Value Health.* 2018;21(2):131-139.

15. Institute for Clinical and Economic Review. *2020-2023 Value Assessment Framework.* Institute for Clinical and Economic Review; October 23 2020.

16. Buermeyer N, Engel C, Nudelman J, Rasanayagam S, Sarantis H. Paths to Prevention: The California Breast Cancer Primary Prevention Plan. Breast Cancer Prevention Partners. <https://www.bcpp.org/our-work/policy-projects/breast-cancer-plan/>. Published 2020. Accessed 28 January, 2021.

17. Woodruff TJ, Sutton P, Navigation Guide Work Group. An evidence-based medicine methodology to bridge the gap between clinical and environmental health sciences. *Health Affairs.* 2011;30(5):931-937.

18. California Department of Pesticide Regulation. *A Guide to Pesticide Regulation in California 2017 Update.* California Environmental Protection Agency. 2017.

19. California Department of Toxic Substances Control Safer Consumer Products Program. *Safer Consumer Products: Alternative Analysis Guide Version 1.1.* 2020.

20. California Office of Environmental Health Hazard Assessment. What We Do. OEHHA. <https://oehha.ca.gov/about/what-we-do>. Published 2020. Accessed 27 April 2021.

21. National Research Council. *Review of California's Risk-Assessment Process for Pesticides.* Washington, DC: The National Academies Press; 2015.

22. Lam J, Koustas E, Sutton P, et al. The Navigation Guide - evidence-based medicine meets environmental health: integration of animal and human evidence for PFOA effects on fetal growth. *Environ Health Perspect.* 2014;122(10):1040-1051.

23. Woodruff TJ, Sutton P. The Navigation Guide Systematic Review Methodology: A Rigorous and Transparent Method for Translating Environmental Health Science into Better Health Outcomes. *Environmental Health Perspectives.* 2014;122(10):1007-1014.
